# Osteofibrous dysplasia: a narrative review

**DOI:** 10.1186/s13018-024-04682-3

**Published:** 2024-03-27

**Authors:** Rui Liu, Linjian Tong, Haiyang Wu, Qiang Guo, Lixia Xu, Zhiming Sun, Hua Yan

**Affiliations:** 1 Clinical College of Neurology, Neurosurgery and Neurorehabilitation, Tianjin Medical University, Tianjin, 300070 China; 2 Duke Molecular Physiology Institute, Duke University School of Medicine, Durham, NC USA; 3 Department of Orthopedics, Baodi Clinical College of Tianjin Medical University, Tianjin, 301800 China; 4 Tianjin Key Laboratory of Cerebral Vascular and Neurodegenerative Diseases, Tianjin Neurosurgical Institute, Tianjin Huanhu Hospital, Tianjin, 300350 China

**Keywords:** Osteofibrous dysplasia, Osteofibrous, Adamantinoma, Fibrous dysplasia

## Abstract

Osteofibrous dysplasia (OFD) is a rare, benign, self-limited bone disorder with a relatively low incidence, accounting for approximately 0.2% of all primary bone tumors. It was frequently found intra-cortical of the mid-shaft of the tibia. OFD can also occur in other skeletal regions, including the fibula, ulna, radius, femur, humerus, ischium, rib, tarsus, metatarsals, vertebral, and capitate. OFD can present with asymptomatic, mass, pain, swelling, deformity, and even pathological fracture. OFD might be misdiagnosed as adamantinoma (AD) and because they are three subtypes origin from the same family of bone tumors and have similar imaging features. Moreover, pathology could provide evidence for an accurate diagnosis of OFD, but misdiagnosis may occur due to small sampling materials. To date, few studies have comprehensively introduced the epidemiology, clinical manifestations, pathogenesis, radiological features, pathology, and treatment for OFD. We herein discuss clinical signs, diagnosis methods, and treatment options of OFD to improve the understanding of OFD, which is helpful for accurate diagnosis and appropriate treatment.

## Introduction

Osteofibrous dysplasia (OFD) is a rare, benign, and self-limited bone disorder [1, 2]. In 1921, Frangenheim reported the first case and named the lesion “congenital osteitis fibrosa” [3, 4]. Later, in 1938, Lichtenstein et al. [5] termed the lesion “fibrous dysplasia” in the classic published literature. Subsequently, in 1966, Kempson and his colleagues described two cases of “ossifying fibroma”, named it such because the lesion resembled fibrous dysplasia [3, 4, 6]. Then, in 1976, Campanacci named the lesion “OFD of the fibula and tibia” due to its histologic resemblance to fibrous dysplasia [4, 6]. Since then, the authors called the lesion OFD [7].

OFD was frequently found intra-cortical of the mid-shaft of the tibia [3, 7–11]. Moreover, the lesions might also be detected in other skeletal regions, including fibula [7, 10, 12], ulna [13], radius, femur [14], humerus [15], ischium, rib [16], tarsus, metatarsals, vertebral bodies, and capitate [17, 18]. OFD could involve multiple bones and is called polyostotic disease in approximately 5% of all cases, while it usually affects one bone and is called monostotic disease in approximately 85% of all cases. Moreover, patients with monostotic lesions are generally asymptomatic and are occasionally found on x-ray images taken for other reasons, such as trauma [11, 19]. Polyostotic OFD might be related to polyendocrinopathy and jagged cafe´-au-lait spots in McCune–Albright syndrome [5, 20]. The pathogenesis of OFD includes genetic mutations, chromosomal structure and number variations, endocrine abnormalities, and bone dysplasia [21].

However, to the best of our knowledge, few studies have comprehensively introduced the epidemiology, clinical symptoms, pathogenesis, imaging features, pathology, and treatment for OFD. Therefore, we reviewed clinical signs, diagnosis methods, treatment options, and microscopic characteristics of OFD to improve the understanding of OFD, which is helpful for accurate diagnosis and appropriate treatment.

## Epidemiology

OFD has a relatively low incidence, accounting for approximately 0.2% of all primary bone tumors [22]. Most authors reported that males have slightly higher OFD incidence than females [23]. However, Park et al. provided a female predilection in their study [4]. OFD is commonly found in infancy and childhood [11, 24, 25], whose ages are often lower than 20 years [4, 6, 22]. Gleason et al. reviewed 16 OFD patients and found that the median age of patients was 9.5 years, and 43.8% of the reviewed cases were younger than six years [23]. OFD frequently occurs in the intra-cortical of the tibia mid diaphysis but uncommonly involves the ipsilateral fibula [3, 7, 11]. The incidence of simultaneous invasion of both tibia and fibula was less than 12% [4, 6, 26], and isolated involvement of the fibula is only 3.8% [4]. In addition, bilateral tibia OFD has only been reported in 2 publications [8, 27].

Many authors believe that the disorder’s progression stops with the reaching of skeletal maturity [11, 12, 24, 26, 28–30]. Furthermore, Campanacci et al. [7] and Nagano et al. [4] propose OFD could regress spontaneously at puberty. Local curettage and excision have 25% recurrence postoperatively [31]. Surgical intervention is an alternative for children with pathological fractures, deforming, and extensive lesions before puberty [23, 26]. In addition, extra-periosteal “shark-bite” resection is the most widely used surgical strategy for a patient with OFD [3, 16]. We summarized the data on the epidemiology, clinical symptoms, treatment, and prognosis of OFD (Table 1).

**Table 1:** Clinical data of the study patients

Authors	Published year	Age/sex	Clinical symptom	Site	Radiological characteristics	Histological features	Treatment	Follow up
Dilogo et al. [32]	2015	8y/M	Severe bowing deformity	Left lower leg	X-ray: bowing, bubbled appearance, intracortical osteolytic lesions, no periosteal reaction; MRI: sclerosis of the internal cortical surface	C-shaped bony spicules with immature bone trabeculae lined with active osteoblasts	Wide excision and MSCs transplantation	84 weeks follow-up without recurrence
Nagano et al. [8]	2017	17y/M	Pain	Right front lower leg	X-ray and CT: osteolytic lesions; Bone scintigraphy: focally increased radiotracer uptake in the bilateral tibia	IHC: expression of glucose transporter 1 (GLUT-1) and hexokinase II	-	-
Teo et al. [10]	2007	Neonate/M	Swelling and deformity	Left lower leg	X-ray and MRI: extensive destructive lesion of the tibial shaft, with dysplastic congenital pseudoarthrosis of the lower fibula.	IHC: cytokeratin positivity	Osteotomy, physeal distraction, and Ilizarov technique	46 months
Jobke et al. [33]	2014	Newborn/-	Swelling and pain	Left lower extremity	X-ray: central intraosseous translucent lesion within the proximal dia-metaphyseal region with circular cortical thinning and expansion with the neo-cortical formation	IHC: cytokeratin positivity	Conservative treatment	9 months
Karol et al. [34]	2005	11.8y/F	Fracture	Left tibial and fibular	X-ray: Well-demarcated anterior cortical lytic lesion with sclerotic border in proximal 1/3 of the tibia	OFD	Closed reduction and application of a cast	Died
Segev et al. [35]	2004	6y/M	Solid tumor and Deformity	Left tibia	X-ray: cystic lesion with a sclerotic reactive rim	OFD	6-10years: close follow-up; 10years: remove the lump; 11years: curetted and treated with cryosurgery, space was filled with PMMA	8 years
Kosuge et al. [36]	2011	11y/M	Deformity	Left leg	X-ray: anterior apex bow to the tibia within which a multilocular lesion with mixed radiolucent and sclerotic foci was seen	OFD	Marginal excision of the lesion	-
Simoni et al. [37]	2011	27y/M	Pain	Right leg	X-ray: a large focal area of cortical thickening. Multiple roundish, radiolucent lacunae, soap bubble appearance, no periosteal reaction; CT: absence of a transitional zone and periosteal reaction. MRI: low signal intensity on both T1WI and T2WI	IHC: cytokeratin positivity	-	-
Yoshida et al. [15]	2018	34y/M	Pain	Left upper arm	X-ray: bone tumor at the humeral shaft	OFD	Curettage, intraoperative anhydrous ethanol therapy, and artificial bone graft	7 years
Abraham et al. [38]	2015	13y/F	Pain and swelling	Right leg	X-ray: eccentric expansile lytic lesion	OFD	Extraperiosteal excision, Autologous free fibular graft, and bone graft substitute	2 years
Gopinathan et al. [39]	2016	14y/F	Pain and swelling	Left collar bone	X-ray: a diffuse periosteal thickening encircling the clavicle extending from its sterna end to lateral third; CT: irregularity and sclerosis of the left clavicle along with heterogeneous ossification. MRI: altered heterogeneous T2 signal within the marrow of the clavicle	IHC: cytokeratin (AE1/AE3 + CK-1) positive	Excision of the lesion	-
Exner et al. [40]	2018	38y/M	-	Left tibia	X-ray: multifocal, partially confluent osteolytic lesions	IHC: vimentin and pan-cytokeratin positivity	Observation	7 years
Goto et al. [13]	2001	15y/M	Pain	Right elbow	X-ray: osteolytic lesions in the medial part of the proximal ulna, with thinning of the cortex and a sclerotic change around the osteolytic lesions	The lesion showed typical zonal architecture; the center of the lesion was predominantly fibrous; In addition, scattered woven bone was rimmed by plump osteoblasts	Five months after the surgery, the tumor recurred. Further surgery was not performed. At age 28 years, the patient had no pain, discomfort, or functional disturbance	13 years
Goto et al. [13]	2001	6y/F	Contusion on the left elbow	Left ulna	X-ray: osteolytic lesions on the dorsal side of the left ulna. The cortex was thin, with medullary sclerosis around the lesion	-	Observation	16 years

IHC, immunohistochemistry; OFD, osteofibrous dysplasia; CT, computed tomography; MRI, magnetic resonance imaging; M, male; F, female; MSCs, mesenchymal stem cells; PMMA, polymethyl methacrylate

### Clinical manifestation

OFD can present with asymptomatic, mass, pain, swelling, deformity (anterior bowing of the tibia), and even pathological fracture [11, 24, 37]. Moreover, Gleason et al. [23] found that patients with OFD about 31% had pain, 13% experienced tibial bowing, 19% suffered pathologic fracture, the other 37% were found inadvertently on Imaging that was taken for other reasons, mostly after trauma [26]. Park et al. [4] reported a review of 80 OFD patients and had a similar incidence of each clinical sign compared to Gleason et al. [23]. The physical examination can reveal local tenderness over the tibia.

### Staging

OFD, differentiated adamantinoma (AD), and AD are three subtypes origin from the same family of bone tumors. OFD locates at the benign end of the spectrum, following differentiated AD lies mid-spectrum and AD at the malignant end [31]. Differentiated AD has extremely similar radiological features to OFD, and these neoplasm sub-types cannot be differentiated utilizing x-rays alone. Besides, AD might have more aggressive characteristics.

The total medullary cavity involvement is less frequently found in OFD and differentiated AD [31, 41, 42]. However, the complete involvement of the medullary cavity and cortex could be detected in most AD patients [31, 41, 42]. Besides, soft-tissue involvement and moth-eaten margins have also been described in AD patients [31]. Bethapudi et al. [31] reported that even though small lesions tend to support the diagnosis of OFD and differentiated AD more than AD, the size of lesions is less crucial in differentiating larger lesions [31]. To date, no distinguishing imaging characteristics to differentiate OFD from differentiated AD or AD have been established.

### Radiological characteristics

**Fig. 1:**
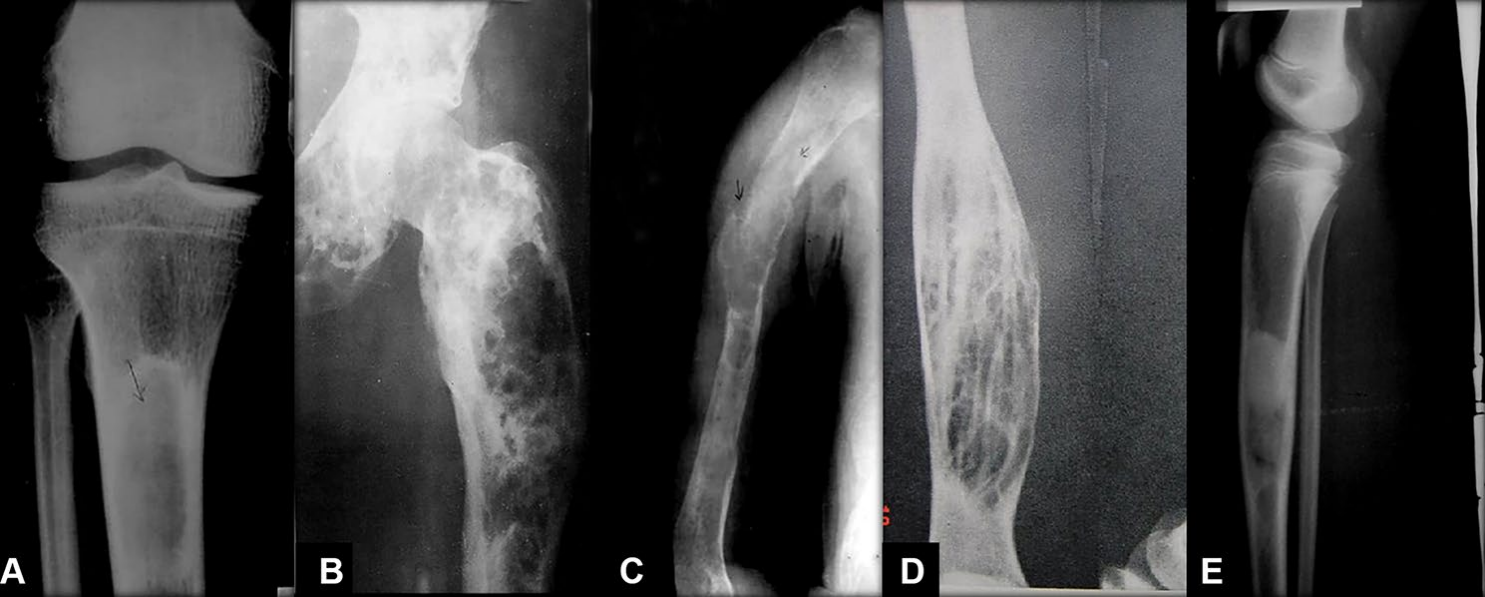
The five subtypes of OFD on radiological. (**A**): ground glass type; (**B**): cystic type; (**C**): insect phagocytic type; (**D**): towel gourd ladle type; (**E**): sclerotic type

**Fig. 2:**
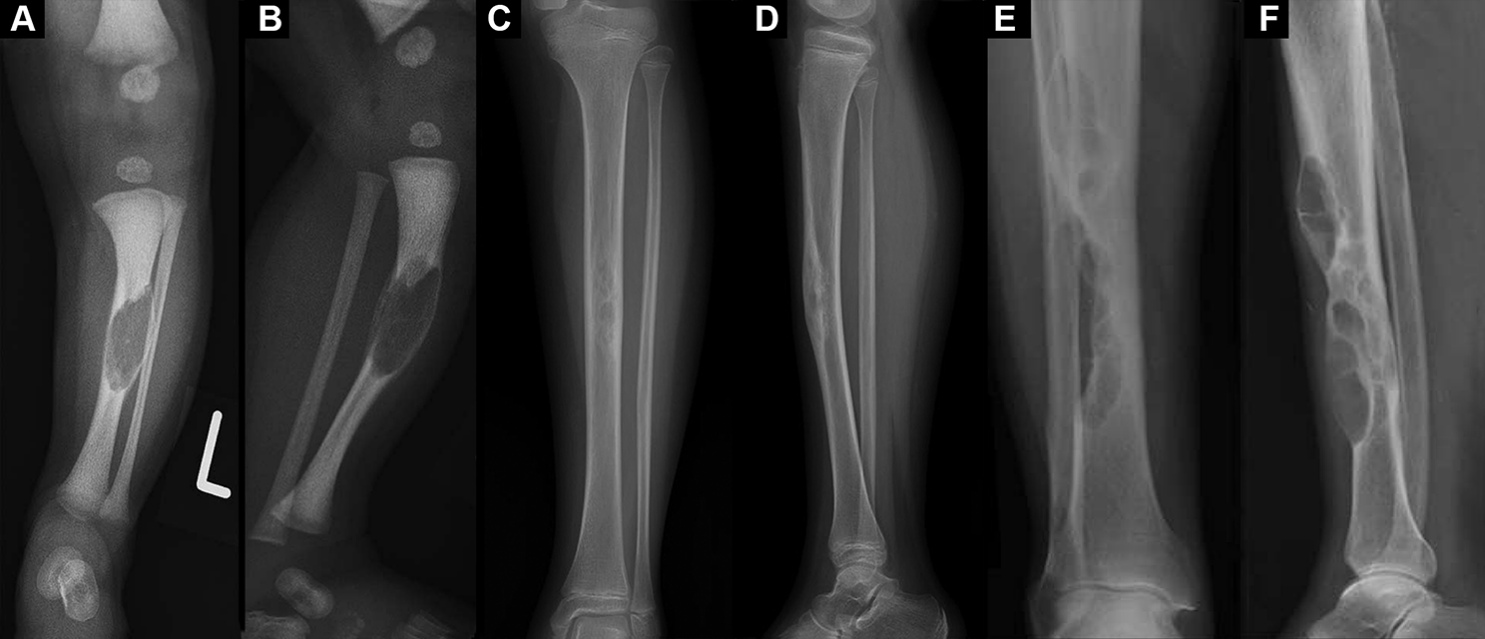
Radiography features of OFD, differentiated AD and AD. (**A**, **B**): A newborn without birth complication and diagnosed with OFD. The left tibia’s frontal and lateral views explain the cortical disruption and oval lucency with cortical thinning. Citation: Jobke B, Bohndorf K, Vieth V, Werner M. Congenital osteofibrous dysplasia Campanacci: spontaneous postbioptic regression. J Pediatr Hematol Oncol 2014, 36(3):249–252. Copyright ©The Author(s) 2022. Published by Baishideng Publishing Group Inc [33]. (**C**, **D**): A 10-year-old girl presented with a 3-month history of pain in her left lower leg after bruising and was diagnosed with differentiated AD. X-rays revealed a 6-cm mass with multiple osteolytic and sclerotic lesions in the thickened anterior diaphysis of the left tibia. Citation: Yamamura Y, Emori M, Takahashi N, Chiba M, Shimizu J, Murahashi Y, Sugita S, Iba K, Hasegawa T, Yamashita T. Osteofibrous dysplasia-like adamantinoma treated via intercalary segmental resection with partial cortex preservation using pedicled vascularized fibula graft: a case report. World J Surg Oncol 2020, 18(1):203. Copyright ©The Author(s) 2022. Published by Baishideng Publishing Group Inc [12]. E-F: a 79-year-old man diagnosed as AD with lower leg pain and an enlarging tibial mass. Multifocal eccentric, expansile lytic lesions are evident, with intervening sclerosis, demonstrating the so-called soap bubble appearance. Citation: Most MJ, Sim FH, Inwards CY. Osteofibrous dysplasia and adamantinoma. J Am Acad Orthop Surg 2010, 18(6):358–366. Copyright ©The Author(s) 2022. Published by Baishideng Publishing Group Inc [26]

Regarding radiography features of OFD, it can be divided into the following five subtypes based on radiological characteristics: ground glass type, cystic type, insect phagocytic type, towel gourd ladle type, sclerotic type (Fig. 1A-E). The x-rays in anteroposterior and lateral views are recommended for the affected area. Anterior eccentric lytic, cortical expansion, and intramedullary extension in the tibial were often found (Fig. 2). The tibia’s anterior bowing deformity, even pathological fracture, could also be detected on the tibial x-rays in patients with OFD. Besides, the lytic of OFD predominantly manifests as an intra-cortical lesion with well-circumscribed edges [43] and is sometimes encircled by a zone of sclerosis [4, 6, 11, 44]. Most, M.J et al. [26] indicated that multiple lucencies might be detected between the sclerotic areas and within the cortical bone, and the affected cortex of the tibia might be thickened or expanded, but the periosteal reaction in OFD patients is uncommon. In addition, as the disorder progresses, the lesion might affect the metaphysic and could show a longitudinal spread.

**Fig. 3:**
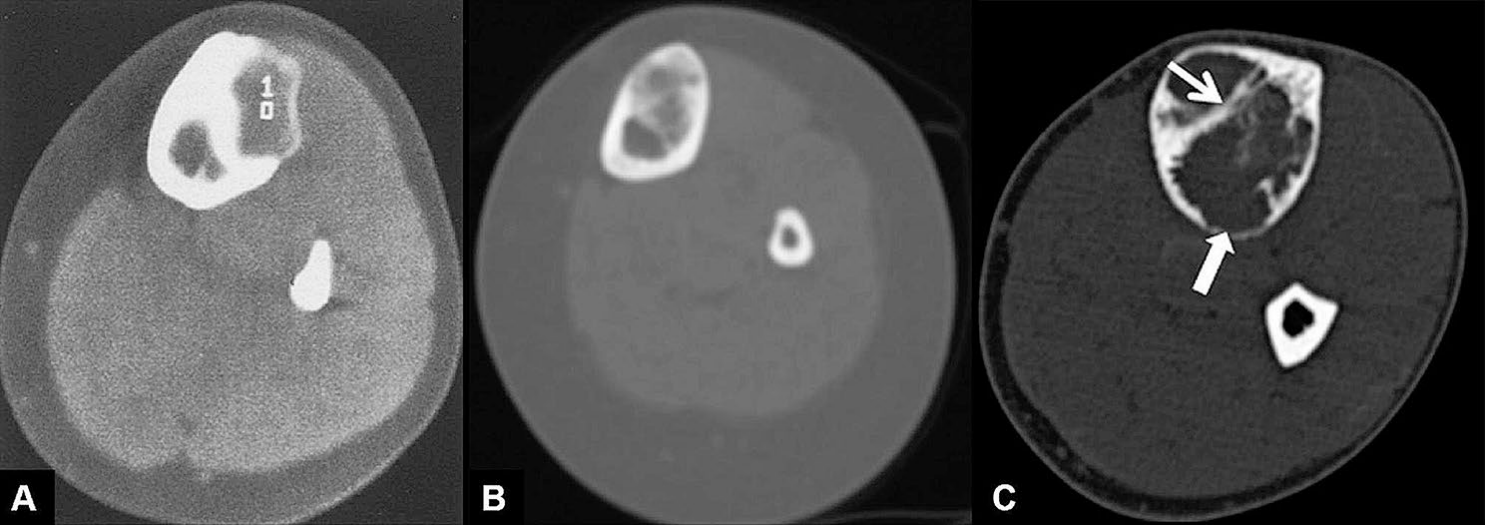
CT features of OFD, differentiated AD and AD. (**A**): CT images of a 14-year-old white female with OFD showed an intracortical expanding lucent lesion with sclerosis of the inner margin. Besides, no intraosseous calcification or soft tissue mass was found. Citation: Ibrahim Fikry Abdelwahab, George Hermann, Joan Zawin, Michael M. Lewis, Klein MJ. Case report 543. Osteofibrous dysplasia of tibia. Skeletal Radiol 1989, 18: 249–251. Copyright ©The Author(s) 2022. Published by Baishideng Publishing Group Inc [75]. (**B**): The CT scans of a 10-year-old girl with differentiated AD revealed that a 6-cm mass was confined to the cortex of the tibia. Citation: Yamamura Y, Emori M, Takahashi N, Chiba M, Shimizu J, Murahashi Y, Sugita S, Iba K, Hasegawa T, Yamashita T. Osteofibrous dysplasia-like adamantinoma treated via intercalary segmental resection with partial cortex preservation using pedicled vascularized fibula graft: a case report. World J Surg Oncol 2020, 18(1):203. Copyright ©The Author(s) 2022. Published by Baishideng Publishing Group Inc [12]. (**C**): The CT images of a 38-year-old man with a classical AD showed obvious destruction of the tibial cortex. Citation: Bethapudi S, Ritchie DA, Macduff E, Straiton J. Imaging in osteofibrous dysplasia, osteofibrous dysplasia-like adamantinoma, and classic adamantinoma. Clin Radiol 2014, 69(2):200–208. Copyright ©The Author(s) 2022. Published by Baishideng Publishing Group Inc [31]

Computed tomography (CT) is better than MRI in evaluating cortical involvement, periosteal reaction, matrix mineralization, as well as pathological fractures (Fig. 3). Nevertheless, CT is only a complement to MRI in the comprehensive evaluation of the lesion but is not a substitute [31].

**Fig. 4:**
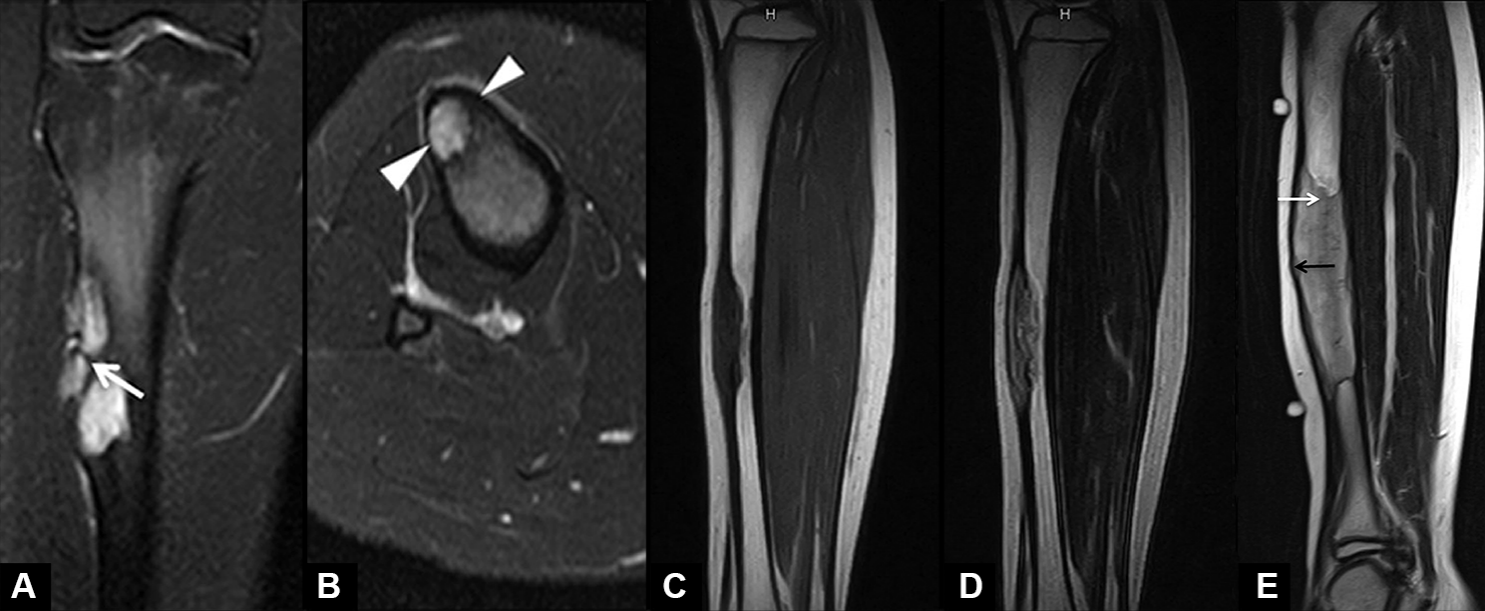
MRI of OFD, differentiated AD and AD. (**A**, **B**): Sagittal and axial T2-weighted fat-suppressed MRI images of a 15-year-old female patient with typical OFD showed that the sclerotic rim has an internal septa (white arrow), and the lesion involves the cortical (triangles) only and not the medullary [31]. The MRI of a 10-year-old girl with differentiated AD illustrated heterogeneously hypointense and isointense on a (**C**) T1WI and heterogeneously hyperintense on a (**D**) T2WI; Importantly, there is an incomplete involvement of the medullary cavity. Citation: Yamamura Y, Emori M, Takahashi N, Chiba M, Shimizu J, Murahashi Y, Sugita S, Iba K, Hasegawa T, Yamashita T. Osteofibrous dysplasia-like adamantinoma treated via intercalary segmental resection with partial cortex preservation using pedicled vascularized fibula graft: a case report. World J Surg Oncol 2020, 18(1):203. Copyright ©The Author(s) 2022. Published by Baishideng Publishing Group Inc [12]. E: Sagittal T1-weighted postcontrast MRI of a 10-year-old male child with a classic AD demonstrated an expansile lesion in the mid-tibial diaphysis and a thinning of the cortex (black arrow); Moreover, it explained complete medullary infiltration (white arrow). Citation: Bethapudi S, Ritchie DA, Macduff E, Straiton J. Imaging in osteofibrous dysplasia, osteofibrous dysplasia-like adamantinoma, and classic adamantinoma. Clin Radiol 2014, 69(2):200–208. Copyright ©The Author(s) 2022. Published by Baishideng Publishing Group Inc [31]

Concerning magnetic resonance imaging (MRI) findings of OFD, numerous authors have reported the MRI appearance of OFD, and they found that the OFD has the characteristic of an osteolytic lesion with a bubbly appearance and lobular loculations with well-circumscribed sclerotic edges [23, 31, 41, 45]. On MRI, Bethapudi et al. [31], Tehranzadeh et al. [41], and Utz et al. [45] found that OFD frequently involves the anterior diaphyseal cortex of the tibia or fibula with adjacent cortical expansion (Fig. 4A-E). Besides, anterior bowing deformity of the tibial diaphysis and intramedullary involvement are frequent complications as the disorder progresses [10, 16]. On MRI, the signal intensity of OFD shows intermediate to high on T2WI and intermediate on T1WI. Multiple factors can affect the signal intensity of OFD in MRI. Firstly, cystic, hemorrhagic, and even cartilaginous differentiation might influence the signal intensity and lead to heterogeneous signal intensity. Secondly, collagen density, the cells, and the degree of mineralization in the osteoid matrix could also affect the signal intensity. Moreover, the imaging features of OFD are similar to other fibroblastic stromal tumors that do not always show such different signal intensity patterns and can present a comparatively well-enhanced pattern that is likely to reflect rich fibrovascular stroma [45]. Thus, MRI can provide some evidence, but it is not the gold standard for diagnosing OFD. Accurate diagnosis directly affects treatment decision-making and prognosis in patients with OFD. Unilocular OFD in images needs to be differentiated with osteoid osteoma, intra-cortical abscess, and intra-cortical hemangioma. Meanwhile, multilocular OFD in images must be differentiated with AD, an aneurysmal bone cyst, osteoblastoma intra-cortical, and fibrous dysplasia.

### Pathogenesis

As for cytogenetic of OFD, the authors analyzed the specimens of patients with OFD and found trisomy abnormalities on chromosomes 7, 8, 12, 21, or/and 22 [46]. Kanamori et al. [47] and his colleagues described the extra copies abnormalities on chromosomes 7, 8, 12, 19, or/and 21 in 2 of 3 patients with differentiated AD and 7 of 8 patients with classic AD. These cytogenetic studies show that OFD might be a clonal tumor lesion rather than a developmental dysplasia [46, 47]. The occurrence of AD may require several steps, including clonal chromosome anomalies, growth factors, and receptors, while OFD and differentiated AD have only undergone partly of them [23]. These three disorders are linked and consistent. However, whether one lesion develops or degenerates into another is still controversial.

Concerning the proteomics of OFD, Maki, and Athanasou [48] revealed a frequent expression of numerous proto-oncogenes, including c-jun and c-fos, and bone matrix proteins including collagen IV, laminin, and galectin 3 in both OFD and AD. Some of these proteins are associated with mesenchymal-to-epithelial differentiation, which provides evidence to interpret why the primary bone tumors contain epithelial components. Bovée et al. [49] researched the expression of growth factors in the epithelial and fibrous portions of AD; they concluded that both the epithelial and fibrous components express fibroblast growth factor receptor-1 and fibroblast growth factor-2, but only the epithelial tissue elements express high levels of epidermal growth factor receptor and epidermal growth factor [49]. Furthermore, AD has a higher epidermal growth factor receptor and epidermal growth factor in the epithelial cells than differentiated AD [49]. In addition, the previous studies found that a proliferation marker and the high levels of Ki-67 can be detected in the epithelial component only [26, 49], which indicating that the epithelial part may be related to malignant activity and tumor growth [26]. It also supports the precursor lesion theory because if the lesion develops from benign to malignant, epithelial cells can obtain a higher expression of fibroblast growth factor-2, epidermal growth factor receptor, epidermal growth factor, and a higher proliferative activity [26].

Concerning molecular analysis for OFD, somatic mutations of the guanine nucleotide-binding protein/a-subunit (GNAS) gene might lead to monostotic fibrous dysplasia, polyostotic fibrous dysplasia, McCune–Albright syndrome, and soft tissue myxoma coexisting with fibrous dysplasia [50]. GNAS gene has the function of encoding the a-subunit of the heterotrimeric G (Gsa) protein complex, and it is located on chromosome 20q13.3 [51]. Alman et al. [52] found two key mutations in exon 8 of the GNAS gene. These mutations are the substitutions of Codon 201, resulting in the substitution of arginine by cysteine (R201C) or histidine (R201H). In addition, there are also uncommon cases with replacement of glycine (R201G) [53], leucine (R201L) [54], and serine (R201H) [55]. Scholars have reported a rare case of fibrous dysplasia related to the mutation of exon 9, resulting in the substitution of glutamine at position 227 by arginine, leucine, histidine, or lysine [56]. In dysplastic cells, all mutations cause an increase in Gsa adenylate cyclase activity and lead to excessive cyclic adenosine monophosphate formation [50]. The increase of intracellular cyclic adenosine monophosphate level might lead to the excessive formation of c-fos, resulting in the uncontrolled expression of osteopontin, ultimately leading to the inhibition of the osteoblasts maturation and increase their proliferation [57]. Thus, these factors lead to the formation of immature dysplastic bone. In a retrospective study of 91 patients with fibrous dysplasia and 40 other fibroosseous disorders, Tabareau-Delalande et al. proved that GNAS mutations were specific to fibrous dysplasia among all fibroosseous disorders [58]. However, several fibrous dysplasias were not showing a GNAS mutation in their study regardless of the molecular methods used, including deoxyribonucleic acid sequencing, allele-specific polymerase chain reaction, and high resolution melting analysis. The fibrous dysplasia patients without measurable GNAS mutation might be interpreted by the tumoral mosaicism of fibrous dysplasia, with high proportions of non-mutated cells compared with mutated cells. However, scholars concluded that GNAS mutation detection is best performed on frozen materials. Although the sensitivity is low, it can be an important diagnostic tool, especially for unconventional and morphologically modified fibrosis dysplasia subtypes. Moreover, the detection results of an OFD patient showed the absence of GNAS mutation could not explain the absence of the GNAS mutation in a case of OFD should not exclude the diagnosis of OFD.

### Pathological

In gross specimens, OFD is mainly confined to the cortical bone, and its color is predominantly yellow to white with a gritty, fibrous uniformity. Under the microscope, the osteoblast margin of the woven bone trabeculae is obvious, and the osteoblastic rimming demonstrates characteristics of fibrous dysplasia [4]. The central osteolytic area characterizes it, wherein the tissue is mainly immature and thin woven bone trabeculae and fibrous with some scanty. Gleason et al. [23] described that from the center to the medullary spaces and periosteal, the bone trabeculae became gradually more numerous, lamellar, mature, and larger until they connected and ultimately merged with the bone.

**Fig. 5:**
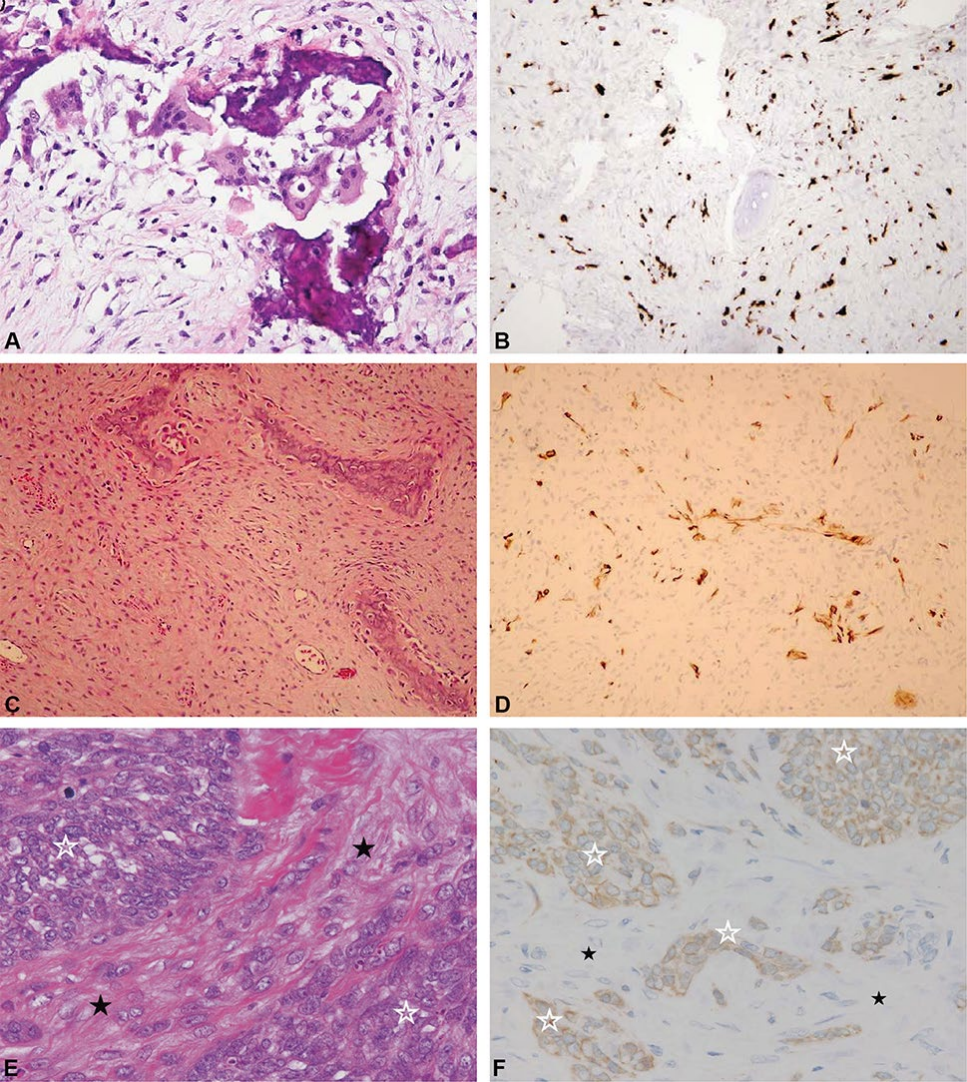
(**A**, **B**): Pathological of OFD; C-D: differentiated AD; E-F: AD. A: Osteoclasts were adjacent to the partially resorbed woven bone; B: Several single keratin-positive stromal spindle cells were detected; Citation: Gleason BC, Liegl-Atzwanger B, Kozakewich HP, Connolly S, Gebhardt MC, Fletcher JA, Perez-Atayde AR. Osteofibrous dysplasia and adamantinoma in children and adolescents: a clinicopathologic reappraisal. Am J Surg Pathol 2008, 32(3):363–376. Copyright ©The Author(s) 2022. Published by Baishideng Publishing Group Inc [23]. C: Hypocellular epithelioid-shaped cells in the osteofibrous tissues were found (×100); D: Epithelium cells stained with keratin (×100). Citation: Buldu H, Centel T, Kirimlioglu H, Dirik Y. Osteofibrous dysplasia-like adamantinoma in a 3-month-old male infant: a case report. Acta Orthop Traumatol Turc 2015, 49(2):210–212. Copyright ©The Author(s) 2022. Published by Baishideng Publishing Group Inc [76]. E: The lesion was composed of spindle cell proliferation with interlacing fascicles (☆) and prominent epithelial islands (☆) with intervening stroma (★) (×100). F: The tumor cells (☆) were positive for AE1/AE3 (pancytokeratin). The intervening stromal cells were not reactive for AE1/AE3 (×100). Citation: Hatori M, Watanabe M, Hosaka M, Sasano H, Narita M, Kokubun S. A classic adamantinoma arising from osteofibrous dysplasia-like adamantinoma in the lower leg: A case report and review of the literature. Tohoku Journal of Experimental Medicine 2006, 209(1):53–59. Copyright ©The Author(s) 2022. Published by Baishideng Publishing Group Inc [61]

The immunohistochemistry (IHC) has to be used in selected patients to detect epidermal-like cells as the number of epidermal-like cells is small and is not visible in the standard hematoxylin and Eosin staining [37]. IHC staining for keratin, an epithelial marker, emphasizes scarce single and strand epithelial cells within a lesion, suggesting OFD. This histological manifestation is extraordinarily similar to fibrous dysplasia; therefore, their names are similar, but fibrous dysplasia usually lacks the unique osteoblast edge of bone trabeculae. According to previous studies, OFD has a loose, frequently storiform fibrous background, including spicules of woven bone trabeculae lined by a layer of osteoblasts [4, 23, 59]. Moreover, OFD reveals a zonal architecture where more immature woven bony trabeculae are located centrally (Fig. 5A-B).

Even in the pathological examination, OFD may still be misdiagnosed as AD. Papagelopoulos et al. [18] have been reported that OFD was initially diagnosed on a small biopsy of the lesions, but AD was ultimately diagnosed following sufficient tissue was taken. Thus, percutaneous or other limited biopsy specimens might lead to sampling errors that should be considered, and a large tissue specimen is recommended for differentiation. Whether OFD can progress to differentiated AD and AD has always been a controversial issue. Initially, Park et al. [4] and Sweet et al. [59] found that no OFD patient progressed to AD during their follow up period. Subsequently, scholars believe that OFD was a precursor lesion that can progress to typical full-blown AD [3, 22, 59, 60]. As the presence of an intermediate lesion, differentiated AD seemed to favor that possibility [61]. Other authors proposed that the differentiated AD indicating a reparative procedure that was the body’s response to a spontaneously regressing AD [23, 62]. However, there is insufficient evidence for regressing AD. Recently, several scholars evaluated a possible common histogenesis between OFD, differentiated AD, and AD (Fig. 5C-F) [12, 19, 24, 29, 31, 60]. With the development of electron microscopy and IHC technology, the AD has been proved to originate from the epithelial cell [17, 20], which rises how epithelial tumors constitute primary bone tumors. Some scholars consider that the epithelial rests are traumatically implanted into the bone at the time of injury due to approximately 60% of AD patients have an injury preceding diagnosis [63]. Others believe that the neoplasm arises from epithelial cells implanted during embryonic development [6, 63]. Moreover, the differentiated AD lies mid-spectrum with malignant potential, although most remains benign [31]. The differentiated AD differs from AD in its entirely intra-cortical location, earlier presentation (younger than 20 years), and predominance of an OFD-like stroma, particularly with only scarce epithelial cells [23, 62]. Besides, AD presented with OFD-like foci on imaging was also reported [64] (Table 2).

**Table 2:** Differentiation of OFD from adamantinoma and fibrous dysplasia

Items	OFD	AD	FD
Age (years)	10 ~ 20	OFD-like AD: 10 ~ 20 Classic AD: 30 ~ 40 Dedifferentiated AD: ~20	10 ~ 30 [65]
Nature	Benign	Biphasic	Benign
Common location	(1) Tibia and/or fibula (2) Arising within the anterior cortex of the diaphyseal [4, 23]	(1) Tibia and/or fibula, followed in the humerus, ulna, and radius (2) Almost exclusively in the anterior cortex of the diaphysis [62]	(1) Femur and craniofacial bones (2) Arising within the medullary canal [62]
Clinical presentation	(1) Pain (25%~50%) or painless (2) Bony deformities (bowing) (3) Pathologic fractures [4, 23]	(1) Pain and swelling (19%) (2) A palpable mass (3) Bony deformities (4) Pathologic fractures (16%~23%) [66, 67]	(1) Swelling (56.7%) (2) Pain or tenderness (35.6%) (3) Pathological fracture (14.4%) (4) Limping (7.8%) (5) Maine cafe ´-au-lait spots [65, 68]
Imaging findings	(1) A single or multiple, variably sized, sharply marginated radiolucencies within the cortex of the tibia or fibula, with a surrounding sclerotic rim (2) An anterior bowing deformity or pathologic fracture may be observed (3) Soft tissue extension is not present, and intramedullary involvement is unusual (4) A CT scan and MRI are useful for confirming the intracortical location of the lesion [69]	(1) OFD-like AD: Like OFD (2) Classic AD and dedifferentiated AD: a) Well-demarcated, lobulated, radiolucent lesion within cortical bone, imparting a “soap bubble” appearance b) Skip lesions and/or multicentric lesions involving the tibia and/or fibula may be present c) May breach the cortex, extending into the medullary cavity or adjacent soft tissues d) A CT scan and MRI are useful for documenting multifocality, cortical destruction, and soft tissue extension [69]	(1) The healthy bone is replaced with a more radiolucent, ‘‘ground-glass’’ appearing pattern, with no visible trabecular pattern (2) The periosteal surface is smooth and nonreactive (3) Shepherd’s crook deformity [65]
Cross features	(1) exclusively intracortical lesion with a tan-gray, solid cut surface and a gritty consistency (2) The perioseum is intact, and the surrounding cortical bone is usually sclerotic and thickened (3) Intramedullary involvement is not typically seen [69]	(1) Solid, well-demarcated, lobulated lesions, with tan-white cut surfaces (2) Centered within the cortex with variable involvement of the medullary space or extraperiosteal soft tissue [69]	(1) A well-circumscribed, tangrey mass that is dense and variably fibrous with a gritty quality (2) May be prominent cyst formation (3) A glassier, blue-tinged appearance may be found in cases with chondroid metaplasia
Microscopic features	(1) A loose storiform, fibrovascular stroma, and woven bony trabeculae with osteoblastic rimming [70] (2) individual keratin-immunoreactive cells can be detected	(1) OFD-like AD: widely scattered, clearly visible small epithelial nests (2) Classic AD: a predominant epithelial component, embedded in an inconspicuous OFD-like bland spindled or fibro-osseous stroma. Besides, the epithelial component forms nests, large anastomosing groups, or sheets of monomorphic cells, displaying tubular or glandular structures, basaloid architecture with peripheral palisading of neoplastic cells, squamous differentiation with associated keratinization (3) Dedifferentiated AD: the abrupt transition of classic AD morphology into a less differentiated, usually pleomorphic sarcoma, losing epithelial differentiation and gaining increased mitoses. Additionally, osteoid or chondroid matrix may be identified in the dedifferentiated foci [71]	(1) It is composed of a bland fibrous stromal proliferation admixed with randomly distributed woven bone (2) Keratin-immunoreactive stromal cells are never observed (3) Nodules of benign hyaline cartilage may be present (4) A key feature is the conspicuous absence of osteoblastic rimming
Immunohistochemical features	Epidermal growth factor receptor was not detected [48]	Epidermal growth factor receptor was detected [48]	Immunohistochemistry serves no purpose in the diagnosis of FD other than to rule out the possibility of a malignant lesion with a pertinent history
Genetic studies	Trisomies of chromosome 7, 8 and 12 [46, 47]	(1) Trisomies of chromosome 7, 8 and 12 (2) extra copies of 19 and/or 21 [46, 47] (3) KMT2D mutations [72] (4) P53 mutations [17] (5) The chromatin remodeling-related gene histone-lysine N-methyltransferase 2D was “recurrently altered” [72]	(1) GNAS mutations [47] (2) Gs-alpha mutations [47]

OFD, osteofibrous dysplasia; AD, adamantinoma; FD, fibrous dysplasia; CT, computed tomography; MRI, magnetic resonance imaging; KMT2D, lysine (K)-specific methyltransferase 2D; P53, tumor protein p53; GNAS, guanine nucleotide-binding protein/a-subunit gene, alpha stimulating activity polypeptide 1

### Treatment

**Fig. 6:**
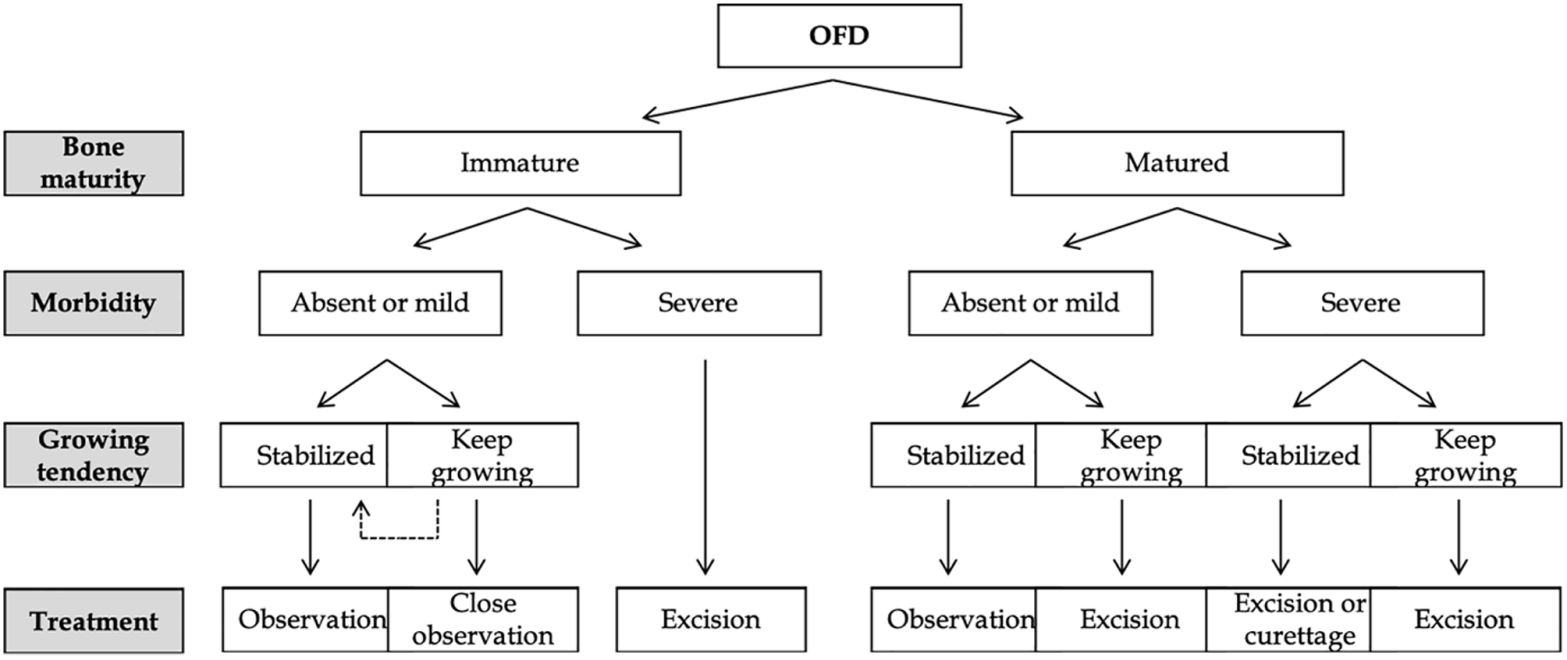
A management strategies for OFD. The main factors in selecting a treatment method include bone maturity, morbidity, and growing tendency. OFD indicates osteofibrous dysplasia. Citation: Park JW, Lee C, Han I, Cho HS, Kim HS. Optimal Treatment of Osteofibrous Dysplasia of the Tibia. J Pediatr Orthop 2018; 38(7): e404-e410. Copyright ©The Author(s) 2022. Published by Baishideng Publishing Group Inc [9]

Currently, the standard treatment recommendations for OFD are challenging to establish due to the low incidence of OFD. According to previous studies, the treatment strategies for OFD include conservative management and surgical intervention [8, 9, 11, 12, 15, 19, 24, 25, 27–30, 40, 43, 44]. Before treatment decision-making, many factors should be considered, including bone maturity, morbidity, and a growing tendency of the tumor [9]. Park et al. [9] presented a treatment algorithm based on the key factors of treatment options for OFD (Fig. 6).

Conservative treatment, including clinical observation [11], patient education [11], and bracing [73], are the main treatment approach for asymptomatic or mild symptomatic OFD patients regardless of bone maturity. Westacott et al. [11] studied 25 OFD patients with the mean age of 6 years and average follow-up duration of 8.3 years, they found that the majority of patients with OFD in tibial can achieve good clinical outcomes and patient satisfaction *via* a less aggressive approach, and the minority of pediatrics need surgical intervention to re-establish stability without removing the lesion. During clinical observation, orthopedic surgeons should cautiously detect all cases with OFD and be more careful with the patients who have symptoms for the first time [9]. Besides, OFD patients with local deformity have the risk of pathological fracture. Thus, bracing can be used to prevent fracture and minimize local deformity.

Surgical interventions including curettage, excision, and extra-periosteal resection are reserved for severe morbidity OFD patients with persistent pain, recurrent fractures, and deformity [23]. Furthermore, in adults, either curettage or excision can be an option for a stable lesion. However, wide excision is advocated for growing lesions due to the risk of lesion developing into malignancy. In the young population, curettage can be carried out only in a limited number of patients, and excision is preferred to prevent disease relapse. Surgery is required in the minority of cases. In addition, if the patient is too young to undergo large excision and reconstruction immediately, curettage can be performed to reduce the size of the lesion and delay the time of definite operation, but it increases the risk of relapse [9]. Other authors reported that any progression of the lesion in OFD comes to an end after puberty, and they also advocated that surgical intervention should be delayed for as long as possible and should be limited to extensive lesions [13].

The reason for the decrease of recurrence rate in older patients after curettage remains unclear. Nevertheless, this finding consists of the behavior of other benign bone tumors, including simple bone cyst, non-ossifying fibroma, and fibrous dysplasia [9]. These benign bone tumors have fewer deterioration characteristics after the growing period [21].

### Prognosis

OFD always has an excellent prognosis because it is a benign lesion in nature; the lesion generally disappears and does not induce other harmful complications in most adulthood. Moreover, there is an association between the benign lesion of OFD and the malignant lesion of AD. Previous scholars have described that most OFD patients with excellent follow-up do not progress to AD ^26^. The minority of OFD cases have described the progression of OFD to AD, but this can be interpreted as an initial misdiagnosis or biopsy sampling error [48]. However, Hatori et al. claimed that the differentiated AD might be a precursor lesion of AD [61]. Thus, the possibility of progression of differentiated AD to an AD should be kept in mind, especially when the destructive changes are observed on imaging [61]. Consequently, Most et al. recommended that all OFD be treated aggressively due to the risk that OFD might progress to AD and sampling error [26]. Additionally, Oka et al. [74] reported the first case of secondary osteosarcoma associated with OFD, and alerted oncologists that OFD may develop into secondary osteosarcoma during long-term follow-up.

### Limitations

This study was a narrative review without systematic evaluation or meta-analysis because OFD is a rare disease and there are very few high-quality clinical RCT articles, so statistical analysis is challenging. In addition, further analysis is needed when enough high-quality studies are published in the future.

## Conclusion

OFD is a rare, benign, deformity-inducing, and self-limited fibro-osseous disease. Its epidemiological characteristics are that OFD often occurs in children under 20 years of age, in boys more than girls, and usually affects unilateral intra-cortical tibia. Clinical manifestations include asymptomatic, mass, pain, swelling, deformity, and even pathological fracture. Radiological features include eccentric lytic, cortical expansion, intramedullary extension in X-rays, and a bubbly appearance with well-circumscribed sclerotic edges in MRI. Clinical observation is an alternative conservative treatment method for asymptomatic and mildly symptomatic patients, while surgical intervention is usually indicated for larger lesions accompanied by more obvious deformities or functional problems caused by the pathological fracture. Multipoint pathological biopsy and accurate diagnosis are crucial. Moreover, OFD usually has an excellent prognosis.

## Data Availability

No datasets were generated or analysed during the current study.
